# *Thermus thermophilus* Argonaute-based signal amplifier for highly sensitive and specific microRNA detection

**DOI:** 10.3389/fbioe.2023.1221943

**Published:** 2023-07-31

**Authors:** Ziqi Wang, Zitong Wang, Fan Zhang, Lingyi Wu

**Affiliations:** ^1^ State Key Laboratory of Digital Medical Engineering, School of Biomedical Engineering, Hainan University, Haikou, China; ^2^ Key Laboratory of Biomedical Engineering of Hainan Province, One Health Institute, Hainan University, Haikou, China

**Keywords:** Argonaute, miRNA detection, signal amplification, single base distinguish, fluorescence assay

## Abstract

The prokaryote-derived gene defense system as a new generation of nucleic acid detection tool exhibits impressive performance in the field of molecular diagnosis. Prokaryotic Argonaute (Ago) is a CRISPR-associated protein that is guided by a short DNA (gDNA) and then efficiently cleaves gDNA-complementary nucleic acids and presents unique characteristics that are different from the CRISPR/Cas system. However, the application of Ago in biosensing is still relatively scarce, and many properties of Ago need to be further clarified. In this study, we aim to systematically explore the properties of *Thermus thermophilus* Argonaute (TtAgo), including the dependence of TtAgo activity on guide DNA (gDNA) length, substrates’ length, and the position of gDNA complementary region on the substrate. Based on these properties, we constructed an exonuclease III-assisted target-recycled amplification system (exoAgo) for sensitive miRNA detection. The result showed that exoAgo can be used for miRNA profiling with a detection limit of 12.2 pM and single-base-resolution and keep good performance for the detection of complex samples, which indicates that Ago has great application potential in the detection of nucleic acids. In conclusion, this study will provide guidance for further development and utilization of Ago in the field of biosensing.

## 1 Introduction

Prokaryotic Argonaute (pAgo), such as *Thermus thermophilus* Argonaute (TtAgo), is an innate immune system like clustered regularly interspaced palindromic repeats and its associated proteins (CRISPR/Cas) that can precisely act on invasive genetic elements with the guidance of an oligonucleotides (Swarts et al., 2014; Hunt Eric et al., 2021). Various CRISPR/Cas (Cas9, Cas12, Cas13, etc.) systems that can execute DNA/RNA cleavage function at 37°C have been widely used for gene editing and regulation and gene detection due to their transcendental specificity and nucleic acids’ cleavage efficiency (Hille et al., 2018; Kaminski et al., 2021; Xu et al., 2022; Wang and Doudna, 2023). Relatively, many pAgo only perform cleavage activity at high temperatures. For instance, the active temperature range of TtAgo is 65∼85°C (Song et al., 2020), and *Pyrococcus furiosus* Argonaute (pfAgo) is approximately 90°C (Wang F. et al., 2021). The dependence of pAgo on high temperature prevents it from being applied for gene editing or regulation. Still, it could have great potential for *in vitro* diagnosis, which requires precision, simplicity, convenience, and low cost-efficiency, and is hardly limited by temperature conditions.

Typically, Ago has an MID and a PIWI domain for recognizing the 5′-phosphate of guide DNA (gDNA), and a PAZ domain for recognizing the 3′-ends of the gDNA, respectively (Sheng et al., 2017). Co-purified experiments showed that TtAgo can only bind to short ssDNA, 15∼18 nt, while longer ssDNA has no obvious interaction with TtAgo (Swarts et al., 2014). Accordingly, Ago nucleases possess higher stability and lower cost-efficiency than Cas enzymes which are usually guided by a long RNA (Zhou et al., 2022). Ago/gDNA assembly can efficiently recognize and cleave complementary DNA/RNA without unique sequence dependence (Li et al., 2023a). This property endows pAgo with flexible designability and predictable targeting capability. Up to now, pAgo has been applied for plasmid construction (Enghiad and Zhao, 2017), fluorescence *in situ* hybridization (Chang et al., 2019), and a multi-turnover nuclease that can be selectively activated by products from PCR (Song et al., 2020; Li et al., 2023b), LAMP (Wang F. et al., 2021), and EXPAR (Lin et al., 2022) to further amplify the detection signal amplification and improve the specificity. Also, pAgo can be used to hydrolyze wild-type genes before the amplification process to achieve the purpose of enriching rare gene mutations (Lapinaite et al., 2018; Shin et al., 2020). These works of research provide powerful evidence for the application potential of pAgo. Still, the characteristics of Ago require further exploration and clarification.

Herein, the characteristics of TtAgo, including the dependence of TtAgo activity on gDNA’s length, substrates’ length, and the position of gDNA complementary region on the substrate, were further explored. On this basis, we developed a detection system named exoAgo, wherein an exonuclease III (Exo III) assisted target-recycled circuit is designed to activate TtAgo cleavage activity. microRNA, a class of short RNA (∼20–25 nt) that can play a negative regulatory role on mRNA expression at the post-transcription level, and usually serves as potential biomarkers of many diseases such as cancer (Sun et al., 2018; Ma et al., 2022), was selected as a model target. The exoAgo system has dual signal amplification mechanisms. First, the Exo III can digest target-hybridized long gDNA (L-gDNA) into short gDNA (S-gDNA), while releasing the target miRNA, which can hybridize with another long gDNA. Second, one Ago can digest several signal probes, thereby further amplifying the detection signals. Through this design, this method could provide excellent sensitivity and specificity for miRNA detection.

## 2 Materials and methods

### 2.1 Materials

The oligonucleotides used for exoAgo were ordered from Sangon Biotechnology Co., Ltd., (Shanghai, China). The magnetic nanoparticle (MNP) was purchased from Nanjing XFNANO Materials Tech Co., Ltd., (Nanjing, China). The 1-ethyl-3-(3-dimethylaminopropyl) carbodiimide hydrochloride (EDC) and N-hydroxysuccinimide (NHS) were purchased from Sangon Biotechnology Co., Ltd., (Shanghai, China). TtAgo and Exo III were obtained from New England Biolabs Co., Ltd., (Beijing, China). RNase-free water (Takara Biotechnology Co., Ltd.) was used for all of the experiments.

### 2.2 The construction of the signal probe

The signal probe (SP) was constructed by covalently linking the FAM-probe to MNP. The sequence of the FAM-probe is NH_2_-TTTTTTTTTTAGATCACAGATTTTGGGCGGGCCAAACTGCTGGGTG-CGGAAGAGAAAGAA-FAM. Briefly, the -COOH groups on carboxylated MNP (200 μL, 50 μg/mL) surface were activated by EDC and NHS, followed by adding an excess FAM probe (20 μM) and vibrating (600 rpm) the mixture under room temperature overnight. By this means, the FAM-probe was covalently linked with the MNP through the amido bond. The obtained SP was washed with 1× phosphate buffer three times and stored at 4°C for further use.

### 2.3 exoAgo processes

At first, the miRNA target was added into 1× phosphate buffer and hybridized with 250 nM of the L-gDNA through 85°C denaturation for 5 min and slowly annealed to room temperature (2°C/min) in 1× phosphate buffer. Then, 0.05 U/μL Exo III was added to hydrolyze the target-hybridized L-gDNA into the S-gDNA at 37°C for 30 min. After that, 50 nM TtAgo protein was added and the mixture was incubated at 75°C for 15 min in 1× Thermopol buffer (10 mM Na_2_HPO_4_, 1.75 mM KH_2_PO_4_, 137 mM NaCl, 2.65 mM KCl, pH 7.4) to make the S-gDNA assemble with TtAgo protein. Subsequently, 5 μL MNP-functionalized SP was added and incubated at 85°C for 40 min so that the assembled TtAgo/S-gDNA complex can cleave the phosphate diester bond of the SP at the 10-11 position of the S-gDNA complementary region. As a result, the fragments-bearing FAM fluorophore was separated from the MNP, and the fluorescence signal could be detected from the supernatant after the sample was placed on a magnetic rack.

### 2.4 The preparation and assay of the plasma-mimic sample

The blood samples were first centrifuged at 1,600 g for 15 min. Then, the supernatant was further centrifuged at 16,000 g for another 15 min to obtain plasma. After that, 10 times continuously diluted let-7a (from 10 pM to 1 µM) were dispersed into 2% plasma and analyzed by exoAgo.

### 2.5 Statistical analysis

All obtained data are displayed as mean ± standard deviations (mean ± S.D.) of three independent experiments. Statistical analysis was performed with OriginPro 2023 10.0.

## 3 Results

### 3.1 Principle

**FIGURE 1:**
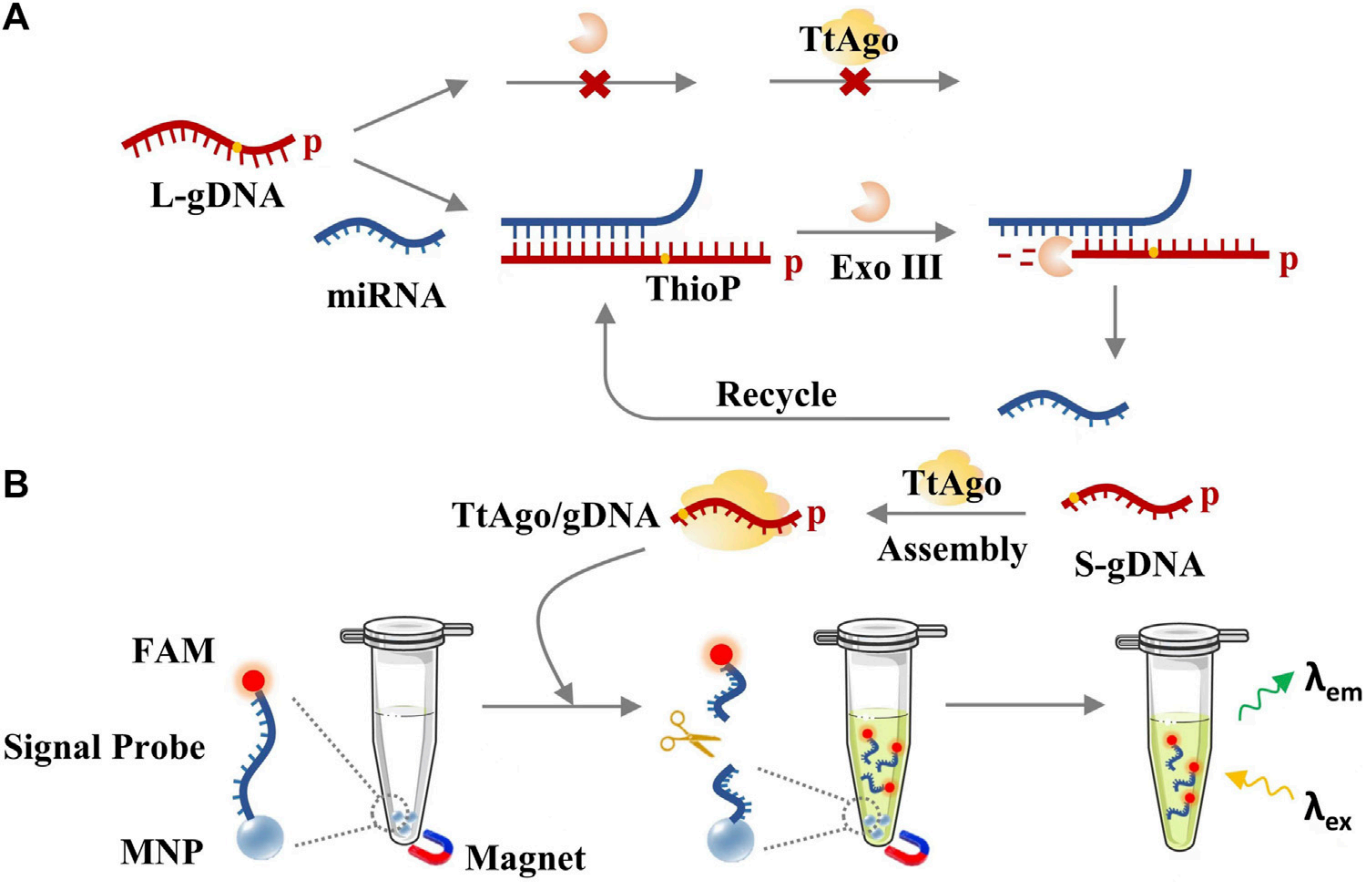
The principle of exoAgo for miRNA detection. **(A)** Exo III-assisted target-recycled circuit. **(B)** TtAgo/S-gDNA-mediated fluorescence detection.

TtAgo can be served as a programmable endonuclease that is guided by a short DNA (∼20 nt) with a phosphate group at its 5′-end. Relatively, long DNA cannot be loaded into TtAgo protein even if it has a 5′-phosphorylated end (Swarts et al., 2014). On this basis, an Exo III-assisted Ago-mediated signal amplification system was designed for miRNA detection. The principle is shown in Figure 1. The L-gDNA with a 5′-phosphate group that was designed to have a complementary 3′-end with the target miRNA cannot directly assemble with the TtAgo protein. After being hybridized with the target miRNA, the miRNA-complementary region of the L-gDNA can be digested by Exo III, which can hydrolyze a single strand in a duplex with a blunt or a 5′-overhang but cannot digest a single-stranded DNA (ssDNA) and a double-strand DNA (dsDNA) with a 3′-overhang (Liu et al., 2020; Wang S. et al., 2021; Lee et al., 2022). In contrast, L-gDNA and Exo III could coexist in the same system without inference in the absence of the target miRNA. Only when the L-gDNA was recognized by the target miRNA and formed a duplex at its 3′-end could Exo III digest its 3′-end to produce TtAgo-available S-gDNA, which could guide TtAgo for further signal amplification. The signal probe was constructed by covalently functionalizing MNP with FAM probes that bearing an S-gDNA complementary region. As a result, activated TtAgo could efficiently cleave the FAM probe, and the signal of the FAM-labeled fragment could leave the MNP surface and be tested from the supernatant, and the fluorescence intensity could be proportional to the target miRNA concentration. Inversely, the sample without miRNA could not obtain cleaved L-gDNA and active TtAgo/S-gDNA complex. Accordingly, the FAM probes would still be linked with the MNP and adsorbed by the magnet, and the supernatant would have no fluorescence signal.

### 3.2 Analysis of TtAgo cleavage features

**FIGURE 2:**
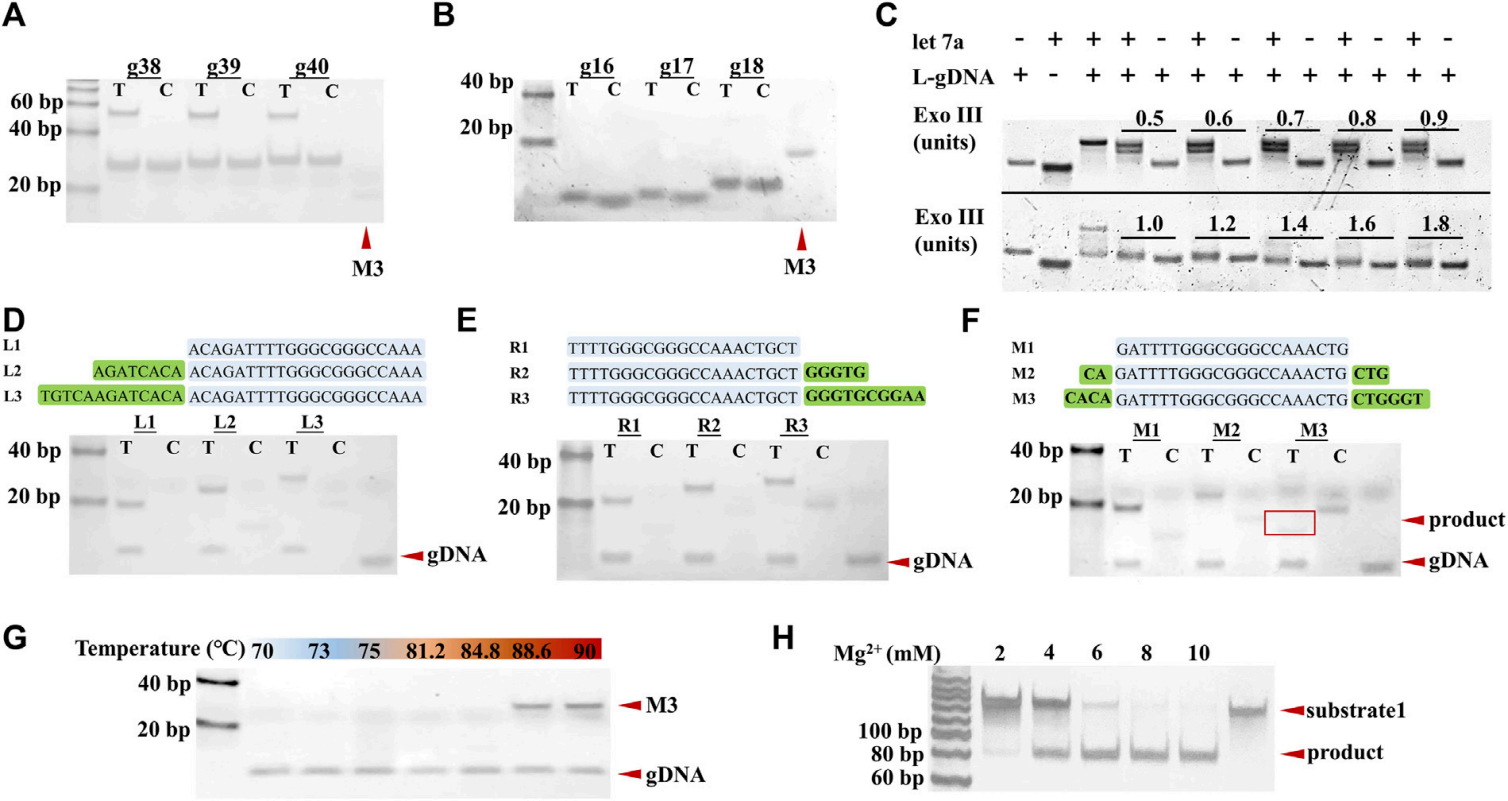
Analysis of the influence factors of TtAgo cleavage. **(A)** PAGE results of L-gDNA-mediated TtAgo cleavage. **(B)** PAGE results of S-gDNA-mediated TtAgo cleavage. **(C)** PAGE results of 1 μM L-gDNA with 1 µM let-7a and various concentrations of Exo III. **(D–F)** Analysis of the effect of substrate’s length and the position of gDNA recognition region on TtAgo cleavage activity by PAGE. **(G)** Analysis of the effect of reaction temperature on TtAgo cleavage activity. **(H)** Analysis of the effect of Mg^2+^ concentrations on TtAgo cleavage activity.

First, the effect of the length of gDNA on TtAgo cleavage activity was verified by polyacrylamide gel electrophoresis (PAGE). As shown in Figures 2A, B, various L-gDNAs with different lengths of 38, 39, or 40-nt, respectively, could not guide TtAgo to cleave substrate, while S-gDNA with lengths of 16, 17, or 18-nt, respectively, could guide TtAgo to efficiently hydrolyze the substrate, of which the sequence is the same as SP. Therefore, the S-gDNA with a length of 16 nt was selected to perform the following experiments. Accordingly, the L-gDNA was designed to have a 16-nt SP complementary region at its 5′-end and a target miRNA complementary region at its 3′-end. To further ensure that the S-gDNA could not be non-specifically cleaved by Exo III, a thiophosphate bond was modified between the 16 and 17 bases of the L-gDNA.

Considering that excess Exo III could cause non-specific DNA cleavage while deficient Exo III could result in a narrow dynamic range, the concentration of Exo III was optimized. Different concentrations of Exo III (from 0.5 U to 1.8 U) were incubated with the L-gDNA and L-gDNA+miRNA, respectively. The effect of Exo III on the L-gDNA was also analyzed by PAGE. The results in Figure 2C show that 1 U Exo III can cleave 1 μM L-gDNA into proper length in the presence of the target miRNA, while more Exo III induced non-specific cleavage of the L-gDNA in the absence of the target miRNA. Thus, 1 U Exo III was used to perform the following experiments.

Subsequently, the effect of the substrate’s length and the position of the gDNA recognition region on TtAgo cleavage activity were also investigated. For these purposes, we designed a series of substrates with various lengths and gDNA complementary region positions and incubated them with TtAgo/S-gDNA under 85°C temperature. The PAGE results are displayed in Figures 2D, E, F. It can be seen that the substrate without extra nucleotides at the left or right side of the gDNA complementary region could not be cleaved by TtAgo/S-gDNA no matter the length. Only the substrate with extra nucleotides at both the left and right side of the gDNA complementary region could be cleaved by TtAgo/S-gDNA. Therefore, it is necessary to consider the length and position of the gDNA complementary region when designing the TtAgo/gDNA substrate.

In addition, the TtAgo reaction conditions, including temperature and Mg^2+^ concentration, were also optimized. The exoAgo was performed under different temperatures (from 70°C to 90°C), and executed with various Mg^2+^ concentrations (from 2 to 10 mM), respectively. From the results in Figures 2G, H, 85°C temperature and 8 mM Mg^2+^ resulted in the highest cleavage efficiency of TtAgo. Therefore, exoAgo was carried out with 8 mM Mg^2+^ under 85°C.

### 3.3 Characterization of the SP

Considering that the cleavage efficiency of TtAgo is significantly affected by the length of the substrate DNA, a typical fluorophore and quencher dual-labeled SP would not be suitable for exoAgo because the quenching efficiency is inversely proportional to the square of the distance between the fluorophore and quencher. The length of SP that should be beyond 31-nt (approximately 10.54 nm) out-of-work distance between fluorophore and quencher is much longer than the effective quenching distance (<10 nm). Also, the molecular beacon is not suitable for exoAgo because its metastable hairpin structure makes it hard to work under the high reaction temperature of TtAgo (85°C). Therefore, we designed a fluorophore (FAM)-functionalized MNP as the SP of exoAgo. In this way, the TtAgo cleavage can be quantified by MNP-mediated separation with the FAM-labeled cleavage fragment.

**FIGURE 3:**
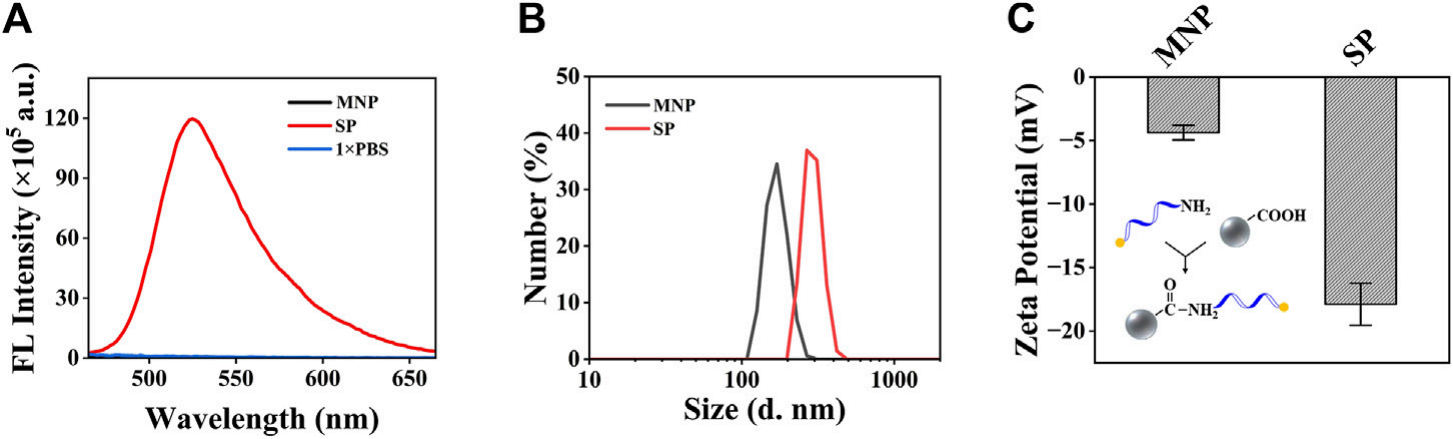
Characterization of the MNP and the SP. **(A)** Fluorescence detection results of the MNP and the SP. **(B)** Analysis of the size distribution of the MNP and SP. **(C)** Analysis of the ζ-potential of the MNP and SP. Both of the plots show mean ± S.D. for three replicates.

To verify the successful construction of the SP, the fluorescence signal, diameter, and ζ-potential of the SP were tested and compared to bare MNP. Before that, BSA was also added to block the site without a FAM-probe on the MNP surface, which may adsorb oligonucleotides non-specifically. The fluorescence detection results (Figure 3A) showed that bare MNP has no fluorescence, while the FAM-probe modified MNP has strong fluorescence signals. Also, it can be seen that the modification of the FAM probe increased the diameter of MNP from 169.9 to 267.2 nm (Figure 3B), and the ζ-potential changed from −4.4 to −17.9 mV (Figure 3C). From these results, it can be confirmed that the SP was successfully constructed.

### 3.4 Analysis of exoAgo performance

**FIGURE 4:**
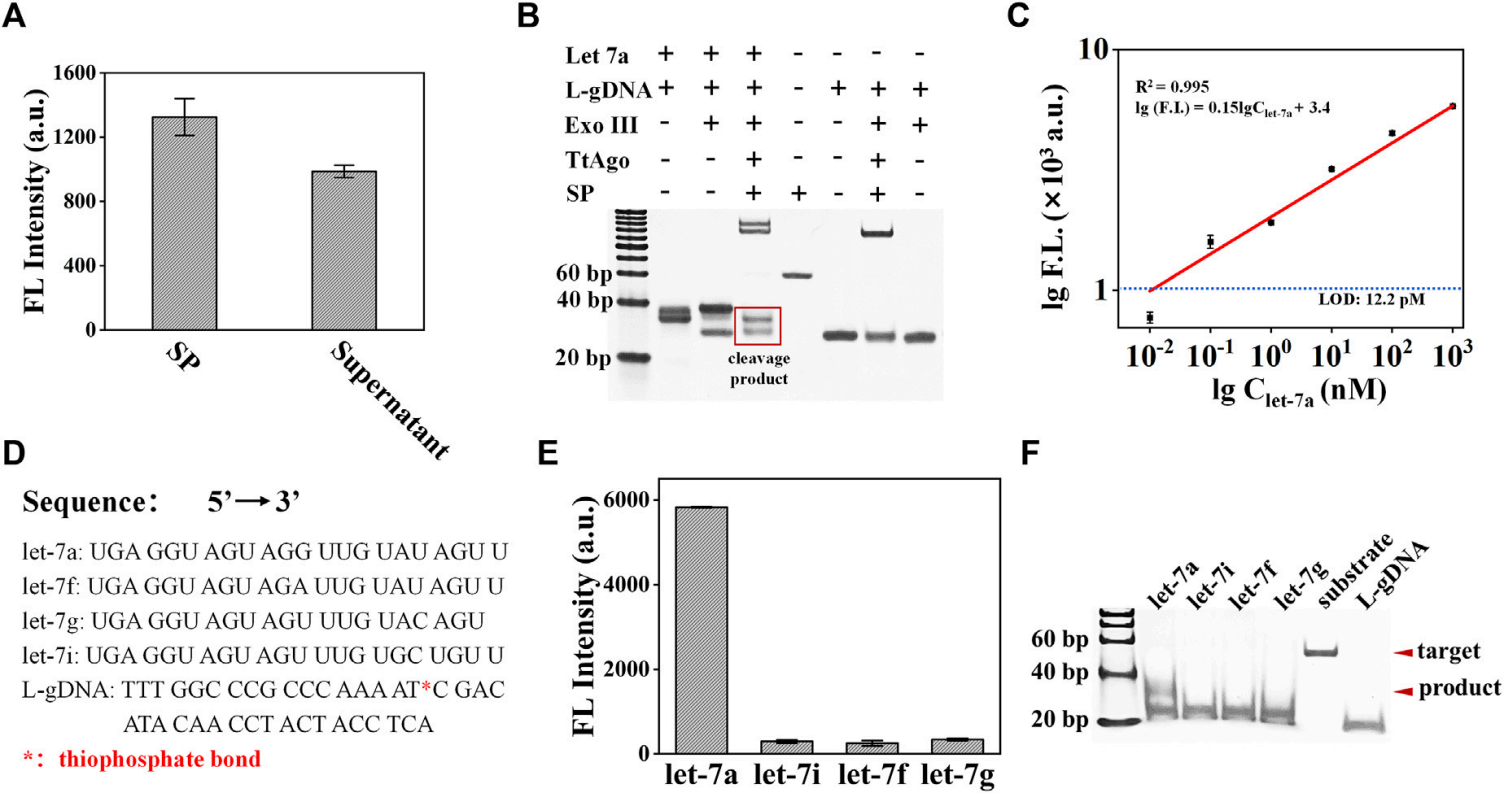
Analysis of the exoAgo performance. **(A)** Analysis of the TtAgo/S-gDNA cleavage ability on the SP by fluorescence detection. **(B)** Analysis of the exoAgo feasibility by PAGE. **(C)** Analysis of the exoAgo sensitivity by detecting let-7a with different concentrations (1 pM, 10 pM, 100 pM, 1 nM, 10 nM, 100 nM, and 1 μM, respectively). **(D)** The sequences of let-7a, let-7i, let-7f, let-7g, and L-gDNA. * represents the thiophosphate bond. **(E)** Analysis of the exoAgo specificity by detection of 1 μM let-7a and other homologous family members (let-7i, let-7f, and let-7g) with the same concentration. **(F)** Specificity analysis of exoAgo by PAGE. All plots show mean ± S.D. for three replicates.

In order to verify the cleavage ability of TtAgo on SP, the TtAgo/S-gDNA complex was directly assembled and incubated with SP under a temperature of 85°C, and the fluorescence signal from the supernatant was detected and compared with the pure SP solution. The fluorescence detection results of the supernatant in Figure 4A confirmed that the TtAgo/S-gDNA can efficiently cleave the SP. Under optimized conditions, we test the feasibility of exoAgo for let-7a. According to the PAGE results in Figure 4B, the exoAgo sample appears to have obvious bands of cleaved SP that are significantly lower than the bands of complete SP, while no cleaved SP bands appear for the blank control sample without let-7a. Therefore, the exoAgo is feasible for miRNA detection.

To analyze the sensitivity of exoAgo, a series of miRNA with different concentrations (from 10 pM to 1 μM) were added into the exoAgo system, respectively. The detection result is shown in Figure 4C. With the increase in the target miRNA concentration, the fluorescence signal also increased. The linear analysis results displayed that exoAgo can achieve a detection limit of 12.2 pM, which is defined as the concentration of the target that yields a net signal equivalent to three times the standard deviation of the blank control sample (*n* = 3) for let-7a detection with a linear equation of lg (FL) = 0.15 × lgC_let-7a_ + 3.4 (*R* ^2^ = 0.995).

Furthermore, we tested the specificity of exoAgo by adding different miRNA (let-7a, let-7i, let-7f, and let-7g) from a homologous family into the exoAgo system, and tested the fluorescence signal of the supernatant. The sequences of let-7a, let-7i, let-7f, let-7g, and L-gDNA with thiophonsphate bond were listed in Figure 4D. The fluorescence detection results in Figure 4E indicate that only let-7a caused a significant fluorescence signal, while the signal of the other three miRNAs that have only 1∼2 base variation with let-7a was negligible. The corresponding PAGE results are shown in Figure 4F. The results confirmed that the exoAgo has a single-base resolution.

### 3.5 Evaluation of the test ability of exoAgo for complex sample

**FIGURE 5:**
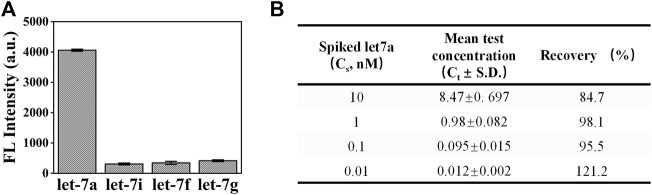
Analysis of the performance of exoAgo for the detection of let-7a in complex samples. **(A)** The detection results of exoAgo for let-7a and the other homologous miRNA (let-7i, let-7f, and let-7g) in diluted plasma. **(B)** Analysis of the recovery (%) of exoAgo for different concentrations of let-7a (0.01, 0.1, 1, and 10 nM) in diluted plasma. Mean represents the average fluorescence intensity. All plots show mean ± S.D. for three replicates.

In order to test the detection performance of exoAgo for complex samples, we first investigated the specificity of exoAgo for plasma samples. A measurement of 1 µM of let-7a, let-7i, let-7f, and let-7g were added into the reaction system. Figure 5A shows that the fluorescence intensity gained from the sample with let-7a was extremely higher than that of other samples. Furthermore, the recovery (%) of exoAgo was evaluated. Different concentrations of let-7a (0.01, 0.1, 1, and 10 nM) were spiked into the diluted plasma sample. We collected the fluorescence intensity data from the supernatant after exoAgo and calculated the test concentration (C_t_) according to the standard curves. The ratio of C_t_ and the actual added concentration of let-7a (C_s_) was defined as the recovery. The results in Figure 5B shows that the recovery of exoAgo fluctuates around 100%, which indicates that the exoAgo possesses good reproducibility.

## 4 Conclusion

In summary, we systematically explored various effect factors on TtAgo activity, including the design of the gDNA, the length, and the corresponding position to the gDNA of the TtAgo cleavage substrate. The experimental results showed that excessively long gDNA could not guide the TtAgo cleavage reaction, and the cleavage substrate had extra nucleotides at either side of the gDNA complementary region. According to these results, we further constructed an exoAgo system for miRNA detection. The exoAgo could provide dual signal amplification: one is the Exo III-assisted target recycle and the other is the TtAgo-mediated cleavage of the signal probe. As a result, the sensitivity of exoAgo for miRNA can reach the pM level and dramatically distinguish the target miRNA from its highly homologous family members with a single base variation from the target. We believe that our exploration of TtAgo characteristics and the scheme of exoAgo for miRNA detection would provide guidance for the development of Ago-based biosensors. With the further discovery and development of Ago proteins, Ago-based signal amplifiers will become more powerful tools in molecular diagnosis.

## Data Availability

The original contributions presented in the study are included in the article/Supplementary Material, further inquiries can be directed to the corresponding author.
