# Supplementary material for: Thermus thermophilus Argonaute-based signal amplifier for highly sensitive and specific microRNA detection
Source: Front Bioeng Biotechnol. 2023 Jul 31;11:1221943. doi: 10.3389/fbioe.2023.1221943 (PMC10424790; doi:10.3389/fbioe.2023.1221943)
Supplement: Supplementary file 1 [file Table1.docx]

Supplementary Material

# Supplementary Tables

Table S1: Synthetic oligo-DNA sequences for this study^§^.

| Name | Sequence (5’-3’) |
| --- | --- |
| let-7a | UGAGGUAGUAGGUUGUAUAGUU |
| let-7i | UGAGGUAGUAGUUUGUGCUGUU |
| let-7f | UGAGGUAGUAGAUUGUAUAGUU |
| let-7g | UGAGGUAGUAGUUUGUACAGU |
| g16 | p - TAACTATACAACCTAC |
| g17 | p - TAACTATACAACCTACT |
| g18 | p - TAACTATACAACCTACTA |
| g38 | p - TTTGGCCCGCCCAAAAAACTATACAACCTACTACCTCA |
| g39 | p - TTTGGCCCGCCCAAAATAACTATACAACCTACTACCTCA |
| g40 | p -TTTGGCCCGCCCAAA ATCAACTATACAACCTACTACCTCA |
| gmps39 | p -TTTGGCCCGCCCAAAAT*CGACATACAACCTACTACCTCA |
| gDNA | p-TTTGGCCCGCCCAAAAT |
| R1 | TTTTGGGCGGGCCAAACTGCT |
| R2 | TTTTGGGCGGGCCAAACTGCTGGGTG |
| R3 | TTTTGGGCGGGCCAAACTGCTGGGTGCGGAA |
| L1 | ACAGATTTTGGGCGGGCCAAA |
| L2 | AGATCACAGATTTTGGGCGGGCCAAA |
| L3 | TGTCAAGATCACAGATTTTGGGCGGGCCAAA |
| M1 | GATTTTGGGCGGGCCAAACTG |
| M2 | CAGATTTTGGGCGGGCCAAACTGCTG |
| M3 | CACAGATTTTGGGCGGGCCAAACTGCTGGGT |
| substrate | FAM - TTTTTTTTTTcgcagcatgtcaagatcacagattttgggcG  ggccaaactgctgggtgcg - Nh_2_ |
| substrate1 | CGCAGCATGTCAAGATCACAGATTTTGGGCGGGCCAAACTGCTGGGTGCGGAAGAGAAAGAATACCATGCAGAAGGAGGCAAAGTAAGGAGGTGGCTTT A |

^§^ The underlined parts are complementary for hybridization; The p represents phosphorylation; The * represents thiophosphate modification.
